# Pyrosequencing the Midgut Transcriptome of the Banana Weevil *Cosmopolites sordidus* (Germar) (Coleoptera: Curculionidae) Reveals Multiple Protease-Like Transcripts

**DOI:** 10.1371/journal.pone.0151001

**Published:** 2016-03-07

**Authors:** Arnubio Valencia, Haichuan Wang, Alberto Soto, Manuel Aristizabal, Jorge W. Arboleda, Seong-il Eyun, Daniel D. Noriega, Blair Siegfried

**Affiliations:** 1 Departamento de Producción Agropecuaria, Universidad de Caldas, Manizales, Colombia; 2 Department of Entomology, University of Nebraska, Lincoln, NE, 68583, United States of America; 3 Universidad del Atlántico, Barranquilla, Colombia; 4 Center for Biotechnology, University of Nebraska, Lincoln, NE, 68588, United States of America; 5 Programa de Biología, Universidad de Caldas, Manizales, Colombia; 6 Entomology and Nematology Department, University of Florida, Gainesville, FL, 32611, United States of America; Institute of Plant Physiology and Ecology, CHINA

## Abstract

The banana weevil *Cosmopolites sordidus* is an important and serious insect pest in most banana and plantain-growing areas of the world. In spite of the economic importance of this insect pest very little genomic and transcriptomic information exists for this species. In the present study, we characterized the midgut transcriptome of *C*. *sordidus* using massive 454-pyrosequencing. We generated over 590,000 sequencing reads that assembled into 30,840 contigs with more than 400 bp, representing a significant expansion of existing sequences available for this insect pest. Among them, 16,427 contigs contained one or more GO terms. In addition, 15,263 contigs were assigned an EC number. In-depth transcriptome analysis identified genes potentially involved in insecticide resistance, peritrophic membrane biosynthesis, immunity-related function and defense against pathogens, and *Bacillus thuringiensis* toxins binding proteins as well as multiple enzymes involved with protein digestion. This transcriptome will provide a valuable resource for understanding larval physiology and for identifying novel target sites and management approaches for this important insect pest.

## Introduction

The banana weevil *Cosmopolites sordidus* (Germar) (Coleoptera: Curculionidae) is considered one of the most invasive and destructive pests of banana worldwide [1]. The larvae of *C*. *sordidus* are a severe constraint on banana and plantain production in most areas where these crops are cultivated, especially in Africa [2–5] where this insect pest has been associated with rapid plantation decline [6] and with a phenomenon called “yield decline syndrome” in West Africa. The larvae of the banana weevil, which are the most destructive stage of the insect, is responsible for considerable damage of the plant corm, interfering with root initiation, nutrient and water uptake and plant development [6]. When a severe weevil infestation occurs, crop losses of up to 100% have been reported [7]. It is well known that chemical control of this insect pest is not only undesirable but also expensive. Options for biological control are limited and pheromone-based insect trapping results in either low or ineffective captures [8, 9].

Many basic advances have been made by studying the banana weevil, including, studies regarding pest resistance [10], insect resistant germplasm [2, 11, 12], plant antifeedants [13], cultural control practices [14] and biological control [15]. Despite extensive and recent biochemical and physiological studies, limited genomic information exists, especially for important tissues such as the midgut. The availability of transcriptome sequences from insect midgut tissues will facilitate identification of genes that are expressed in the intestinal tract and their respective metabolic and functional roles. It is well known that the curculionids are the largest family of beetles [16], which in general are important plant tissue damaging pests such as the banana weevil *C*. *sordidus*[1].

The rapid growth of next-generation DNA sequencing technologies such as 454-based pyrosequencing [17, 18] have allowed the characterization of the transcriptome of many important, non-model insect species [19–23], thus providing valuable and unprecedented opportunities to increase our knowledge of expressed genes, especially in those insect pests where little or no genomic resources exist [24].

In this study, we used a 454-based pyrosequencing platform to sequence the *C*. *sordidus* larval midgut transcriptome allowing the characterization of transcripts encoding different genes associated with metabolic functions and potential insecticide targets. Many of these transcripts were protease-like genes from different digestive enzyme families, mainly associated with aminopeptidases, carboxypeptidases, serine proteases and cysteine proteases. The *C*. *sordidus* transcriptome represents an important contribution to understanding the biology of this insect pest and for the identification of potential target genes involved in protein digestion and many other metabolic pathways.

## Materials and Methods

The experiments were carried out under a standard protocol in the lab and no specific permissions were required for these locations/activities. In addition, these study did not involve any endangered or protected species.

### Insect dissection and of midgut RNA extraction

**Fig 1:**
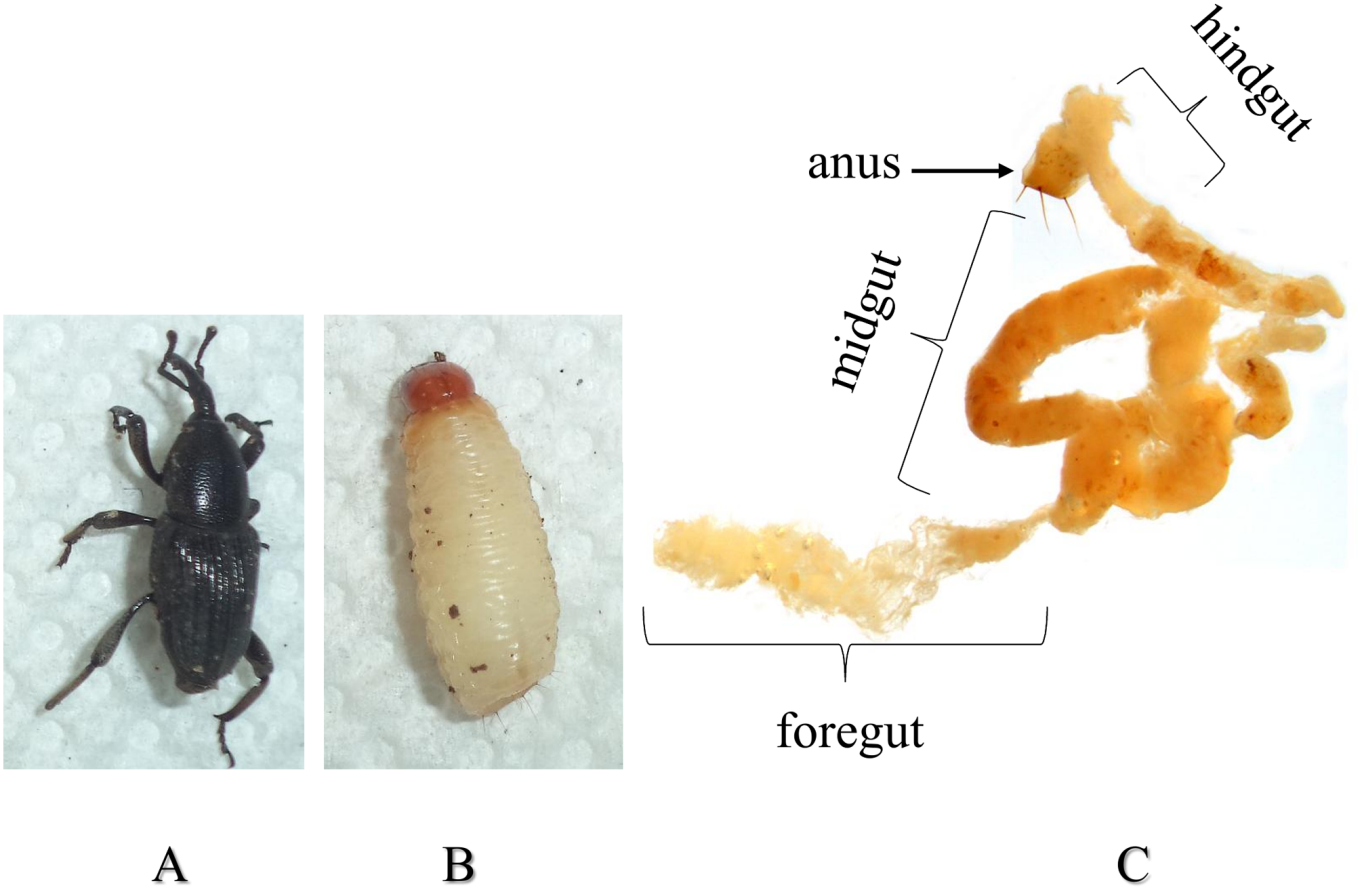
*Cosmopolites sordidus*. Adult (A), fourth instar larva (B), digestive tract from a fourth instar larvae (C).

*C*. *sordidus* larvae were collected from corms obtained at a plantain field near Manizales, Colombia (1058 m, 5° 4’ 13.2” N, 75° 41’ 7.7” O). Collected larvae were inspected under a stereoscope and the fourth instar larvae were selected based on the size of the head capsule as described by [25] and then used for midgut dissection (Fig 1). Gut tissue was obtained by dissecting in DEPC-treated distilled water. The gut content and peritrophic matrix were removed and the washed midgut tissue was flash-frozen using liquid nitrogen and stored at -80°C. RNA extraction was performed using TRIzol reagent (Invitrogen, Carlsbad, CA, USA) following the manufacturer’s instructions. RNA was then purified using the RNeasy MinElute Cleanup Kit (Qiagen, Chatsworth, CA) after removing genomic DNA contamination using the TURBO DNA-*free*^™^ Kit (Ambion, Carlsbad, CA) according to manufacturer’s instructions.

### *C*. *sordidus* midgut normalized cDNA library preparation

Full-length-enriched double-stranded cDNA was then synthesized using the Mint-2 cDNA synthesis kit (Evrogen, Moscow, Russia/ Cat # SK005). To reduce the prevalence of abundant transcripts, the resulting double-stranded cDNAs were normalized using the Evrogen Trimmer-2 cDNA normalization kit (Evrogen, Moscow, Russia/ Cat # NK003) [26]. The resulting normalized cDNA midgut library was then submitted to 454- high-throughput pyrosequencing.

### Sequencing and assembly

For 454 pyrosequencing (Roche Applied Science), 3 μg of normalized cDNAs was sent to the Core for Applied Genomics and Ecology (CAGE) facility at the University of Nebraska-Lincoln. The sequences obtained were preprocessed by filtering reads with low qualities (Q15) that were less than 100 bp as well as trimming SMART adapters and Ns. Finally, processed reads were clustered using the MIRA 3.4.0 assembler.

### Homology searches and sequence annotation

Functional annotation of assembled sequences by gene ontology terms (GO; www.geneontology.org), InterPro entries (InterProScan; http://www.ebi.ac.uk/tools/pfa/iprscan/) and enzyme classification codes (EC) was conducted using Blast2Go software suite [27]. For homology analysis, all sequences were searched against the NCBI non-redundant (nr) protein database via BLASTx using an E-value cut-off of 10^-25^.

### Protein sequence alignment and phylogenetic analysis

The protein sequence of insect carboxypeptidases were aligned with ClustalW program (http://www.ebi.ac.uk/clustalw/). The evolutionary relationship among carboxypeptidases was determined using phylogenetic analysis based on protein sequences and carried out using the Neighbor-joining method using MEGA 6.0 software.

### Semi-quantitative RT-PCR

One microgram of total RNA was used as template for synthesis of the first strand of cDNA with an oligo-(dT) primer and the Maxima H Minus cDNA synthesis kit (Thermo Scientific, kat # K1681)). The cDNA was employed as a template for amplification and detection of Carboxypeptidase (*CsoCp*), Chitin Synthase (*CsoChs*), and Aminopeptidase (*CsoAp*) transcripts in five larval development and pupae stages of *C*. *sordidus*. The expression level of these transcripts was evaluated using a specific set of primers as follow: *CsoCp* sequence (forward primer 5'-CCGAACCTTGCTCTGATACC-3', and reverse primer 5'-CGTACCCCCATGGATACAAC-3'), *CsoChs* sequence (forward primer 5'-CCATTTACCCCGAAGATCAA-3', and reverse primer 5'-TGGATAAACATGCAAATACATTG-3'), and *CsoAp* sequence (forward primer 5'-TTCCTGAATGAGGGATTTGC-3', and reverse primer 5'-GGTGCTTGAAGTGCTTGTGA-3'). *C*. *sordidus* β-actin gene was also amplified by PCR using the following set of primers: forward primer 5'-AAGACATCAGGGCGTAATGG-3', and reverse primer 5'-GAAGGTGTGGTGCCAGATTT-3'. The PCR reaction was carried out in a 10 μl final volume. PCR conditions were: 95°C for 3 min, 60°C for 30 s, 72°C for 30 s followed by 32 cycles, and 5-min extension at 72°C. All PCR products were resolved by electrophoresis on 1% agarose gels.

## Results

### Pyrosequencing, assembly, and annotation

**Fig 2:**
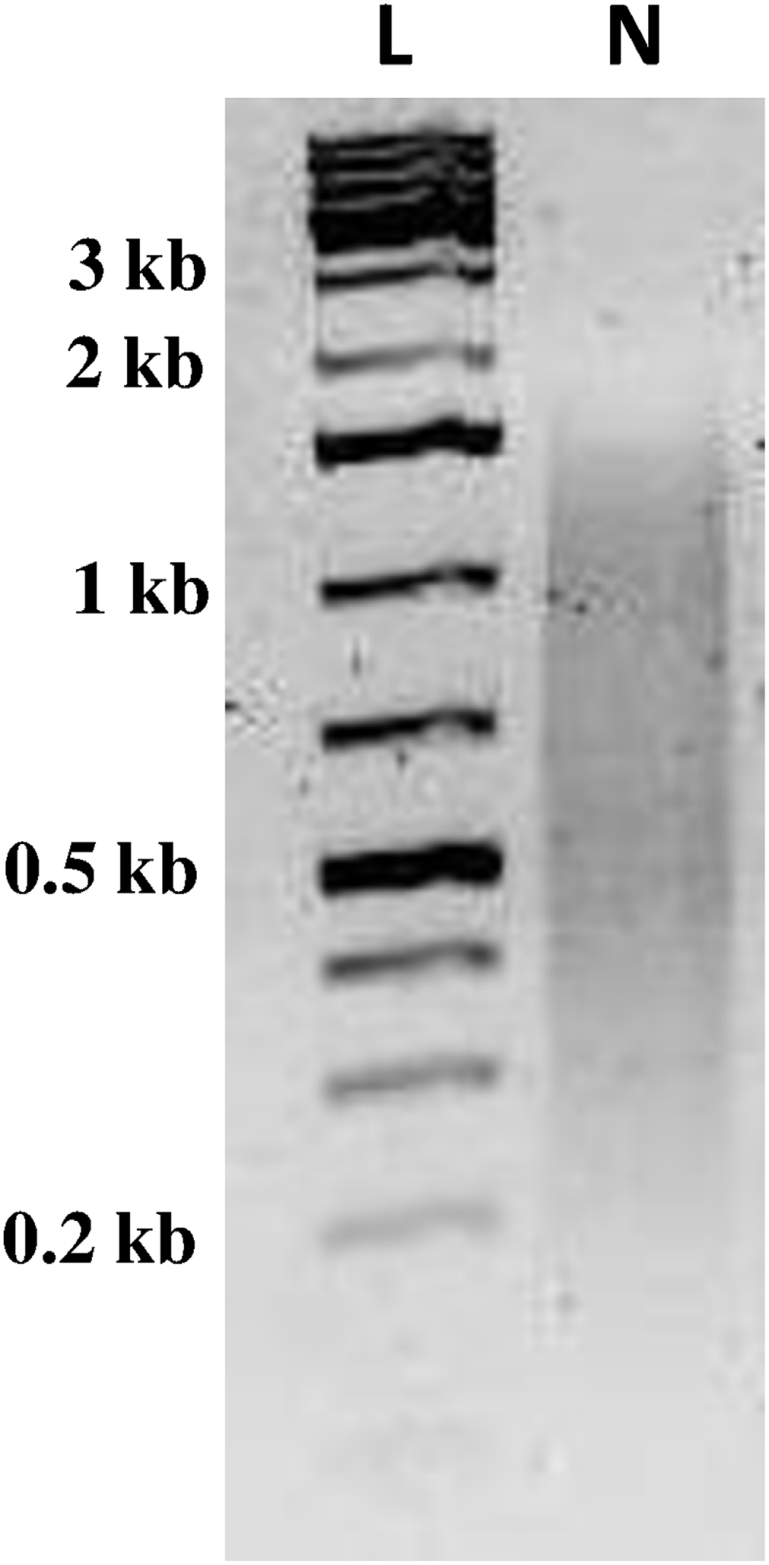
Electrophoresis of normalized cDNA from *C*. *sordidus* midgut. Normalized cDNA library containing transcripts ranging from 0.2 to 1.5 kb in size were subject to 454-mediated pyrosequencing. L, molecular mass markers. N, normalized cDNA library.

Normalization of the *C*. *sordidus* midgut cDNA library resulted in an even distribution of transcripts ranging from 0.2 to 1.5 kb in length (Fig 2). 454-pyrosequencing of the normalized library from C. *sordidus* midgut transcriptome generated a total of 596,389 sequencing reads with an average length of 491 bp (Table 1). After filtering reads with low quality (Q15) and less than 100 bp in length as well as trimming SMART adapters and Ns, 425,605 reads were assembled using the MIRA 3.4.0 assembler. The assembly resulted in 47,729 contigs and 139,600 singletons that did not assemble into a contig. The average contig length was 491 bp (100–4270 bp) with N50 of 505 bp (Table 1). These data were deposited in NIH Short Read Archive with accession number SRP061782. It was found that almost 35% of all contigs returned at least one blast hit and one GO term (Table 1). In addition, 13.5% of these contigs (6,457) received an EC number, which assigned a known enzymatic function.

**Table 1:** Summary statistics for *C*. *sordidus* midgut transcriptome after assembly and annotation.

**Assembly**	
Total number of reads (before filtering)	596,389
Number of reads that entered assembly (after filtering)	425,600
Total base pairs that entered assembly	178,143,660
Average contig length (range)	491 (100–4270)
N50 length (bp)	505
Number of contigs >400 bps	30,840
**Annotation**	
% contigs with at least one GO term	34.4%
% contigs with at least one blast hit	35.4%
% contigs with at least one InterPro cross ref	34.5%
% contigs with an EC number	13.5%

### Functional classifications, homology searches and Gene Ontology Analysis

**Fig 3:**
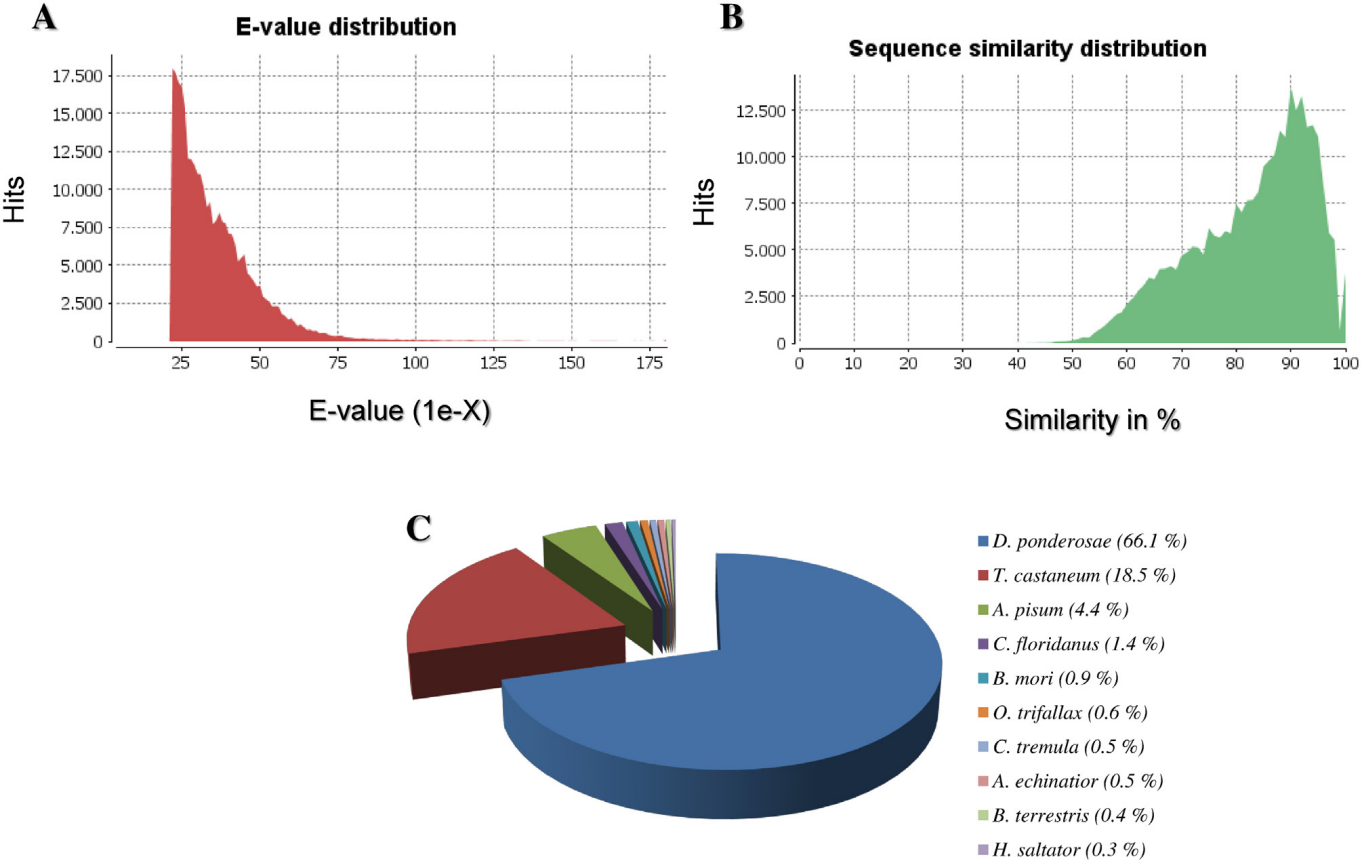
Summary of homology searches (BLAST) of *C*. *sordidus* midgut 454-pyrosequencing data against the non-redundant (nr) database. (A) E-value distribution (Cut-off 10–20). (B) Similarity distribution of the top BLAST hit. (C) Species distribution of the top BLAST hit. Note that the majority of top hits are to the beetles *D*. *ponderosae* (66.1%) and *T*. *castaneum* (18.5%).

**Fig 4:**
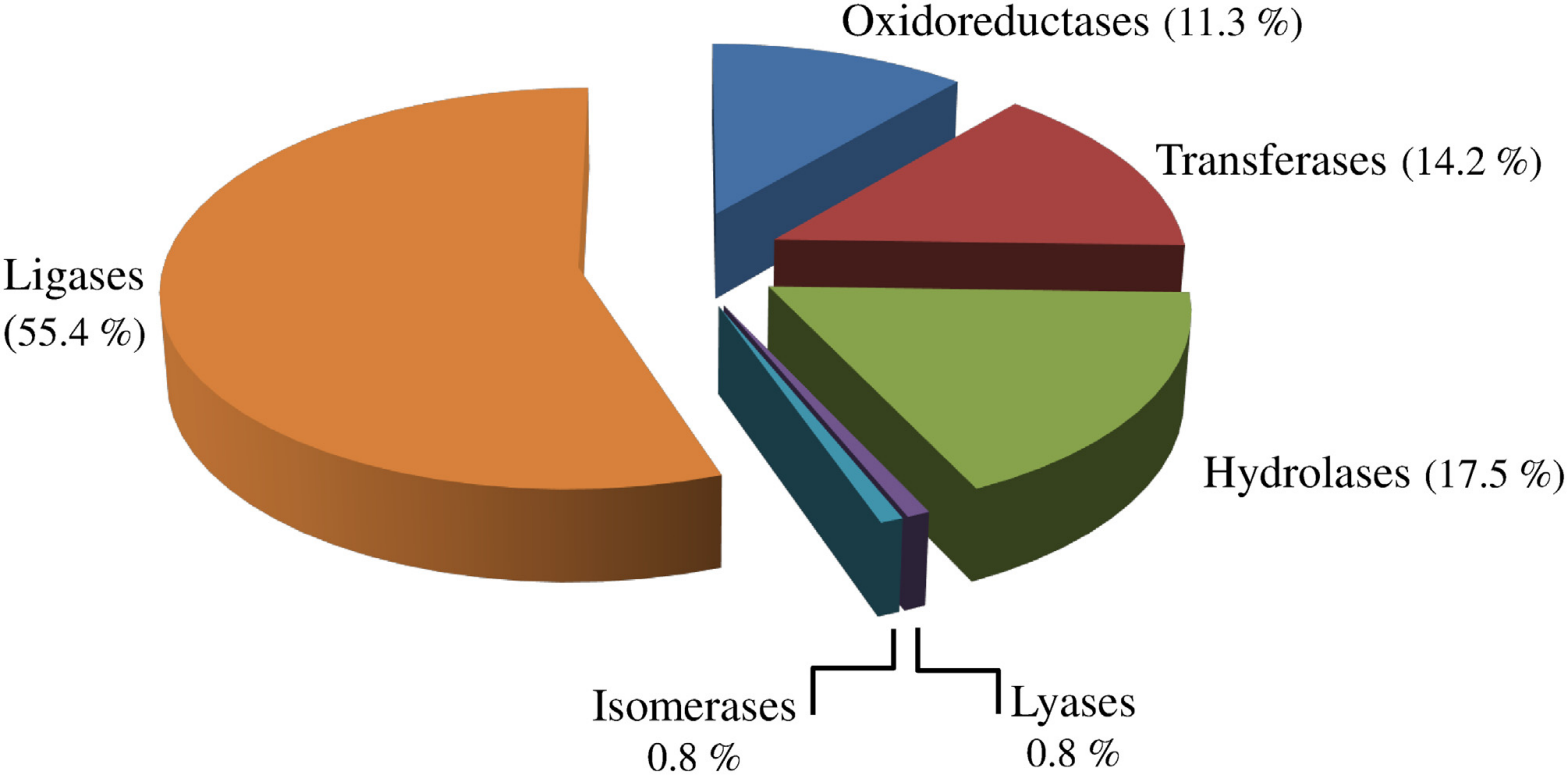
General Enzyme Classification (EC) terms for the contigs of *C*. *sordidus* midgut transcriptome. Oxidoreductases (EC: 1.x.x), Transferases (EC: 2.x.x), Hydrolases (EC: 3.x.x), Lyases (EC: 4.x.x), Isomerases (EC: 5.x.x), and Ligases (EC: 6.x.x).

After read assembly, contigs were submitted to BLASTx similarity search against NCBI non-redundant protein database (nr) to assess their putative function. The similarity distributions and E-value of the *C*. *sordidus* BLAST hits against the non-redundant database are presented in Fig 3. Most of the BLAST hits are to the bark beetle *Dendroctonus ponderosae* (66%) and to the model coleopteran, *Tribolium castaneum* genomes (18.5%) (Fig 3C), which is one of the few beetle genomes that has been fully sequenced so far. Enzyme classification (EC) was used to classify the predicted *C*. *sordidus* midgut proteins. Enzyme classification shows that ligases account for the largest proportion of *C*. *sordidus* enzymes (55.4%), followed by hydrolases (17.5%), transferases (14.2%) and oxidoreductases (11.3%) (Fig 4). In addition to enzyme classification, gene ontology (GO) assignments were used to classify the functions of the predicted proteins, producing 37,982 terms for biological process categories, 16,457 terms for cellular component categories and 22,870 terms for molecular function categories.

**Fig 5:**
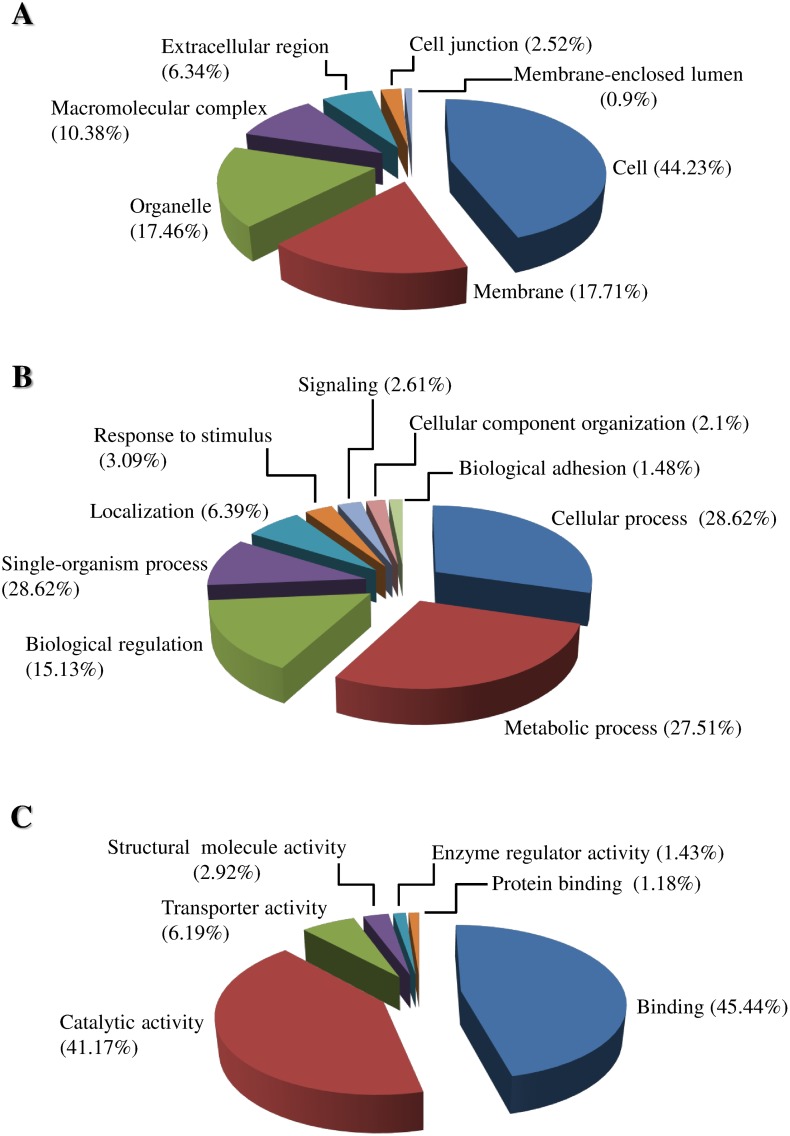
Gene ontology (GO) assignments for the *C*. *sordidus* midgut transcriptome. Panel A represents cellular components, while panel B represents biological processes and panel C is for molecular function at level 2.

**Fig 6:**
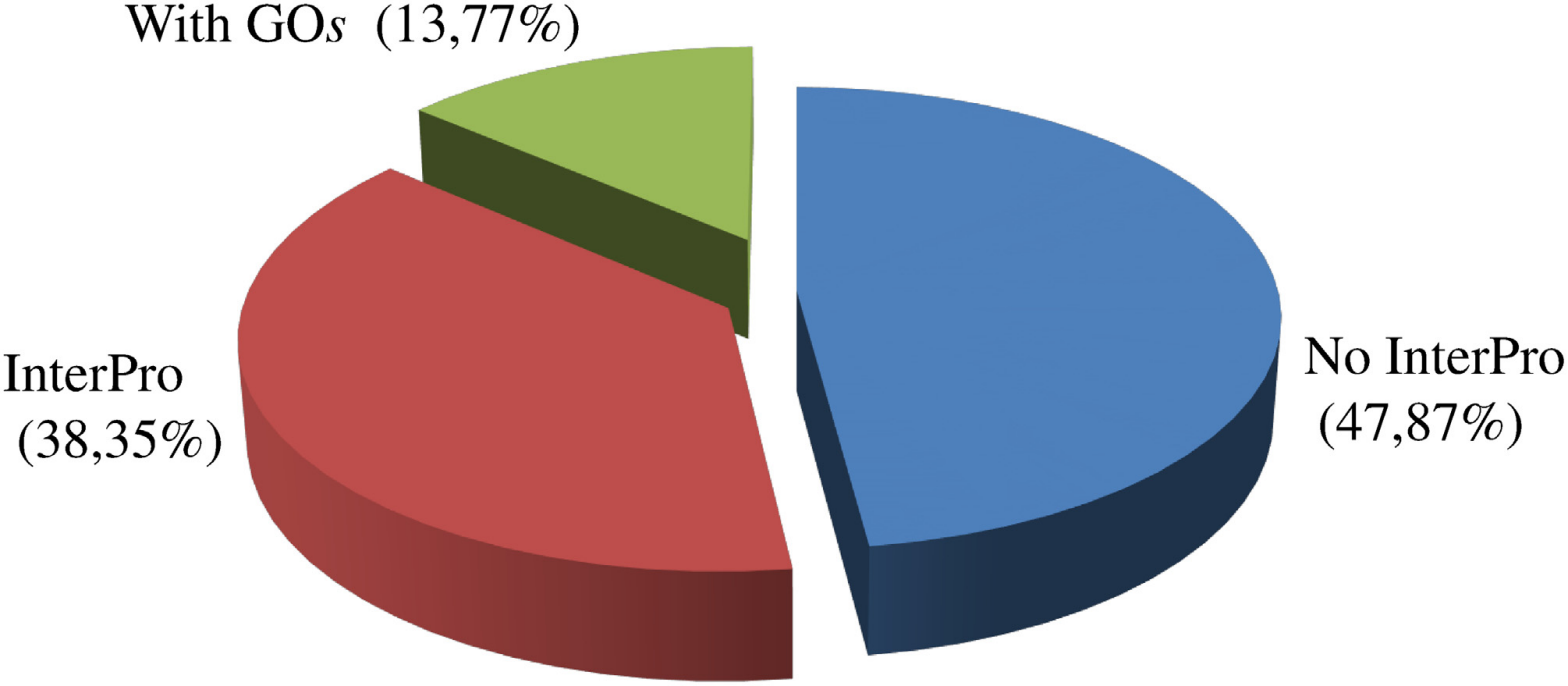
Summary information for InterProScan results in the *C*. *sordidus* midgut transcriptome.

Most of the cellular component GO terms (Fig 5A) were associated with the cell (44.23%) followed by the membrane (17.71%) and organelle (17.46%). Metabolic (27.51%) and cellular processes (28.62%) were involved with more than half of the biological process GO terms followed by biological regulation (15.13%) (Fig 5B). Most of the molecular function GO terms were associated with binding (45.44%) followed by catalytic activity (41.17%) and transporter activity (6.19%) (Fig 5C). The InterPro analysis was also used in addition to enzyme classification and GO assignments and identified that almost 14% of predicted proteins received a GO assignment and almost 48% of the predicted *C*. *sordidus* proteins did not have an InterPro assignment (Fig 6).

### Genes of interest related to midgut metabolic functions and xenobiotic metabolism

A list of *C*. *sordidus* larval genes related to general digestion, peritrophic membrane biosynthesis, degradation and remodeling as well as detoxification and protease-like related genes are presented in Table 2. A total of 51 detoxification related contigs were identified in the *C*. *sordidus* midgut transcriptome. Of these, 22 corresponded to cytochrome P450 genes, 11 to glutathione-S-transferases, 13 to carboxylesterases and 5 to superoxide dismutases (SOD). Contigs related to peritrophic membrane biosynthesis, degradation and remodeling include chitinase (15), chitin synthase (6), and chitin deacetylase (5). Contigs associated with general digestion from this pyrosequencing analysis include cysteine proteinases (143), serine proteinases (61), aminopeptidases (38), carboxypeptidases (22), Dipeptidyl peptidases (8), lipases (3), and β-glucosidases (7). An additional 40 contigs related to immunity and defense against pathogens were identified from the *C*. *sordidus* midgut transcriptome. Among these contigs, lectin (17) and serine protease inhibitors (serpin) (11) were the most abundant.

**Table 2:** Selection of *C*. *sordidus* larval genes related to midgut metabolic functions and “Detox” related.

	EC number	Total number of contigs
**“Detox” related**		
Cytochrome P450	-	22
Glutathione-S-transferase	2.5.1.18	11
Carboxylesterase	3.1.1.1	13
Superoxide dismutase	1.15.1.1	5
**Peritrophic membrane biosynthesis, degradation, and remodeling**		
Chitinase	3.2.1.14	15
Chitin synthase	2.4.1.16	6
Chitin deacetylase	3.1.5.41	5
**General digestion**		
Cysteine proteinase all types	-	143
Serine proteinase all types	-	61
Aminopeptidase all types	-	38
Carboxypeptidase all types	-	22
Dipeptidyl peptidase all types	-	8
Lipase	3.1.1.3	3
β-glucosidase	3.2.1.21	7
**Immunity-related and defense against pathogens**		
Peptidoglycan recognition protein	-	5
C-type lectins	-	17
Defensin-like	-	6
Lysozyme	-	1
Serin protease inhibitors (Serpin)	-	11

### Protein alignment of protease-like enzymes and phylogenetic analysis

**Fig 7:**
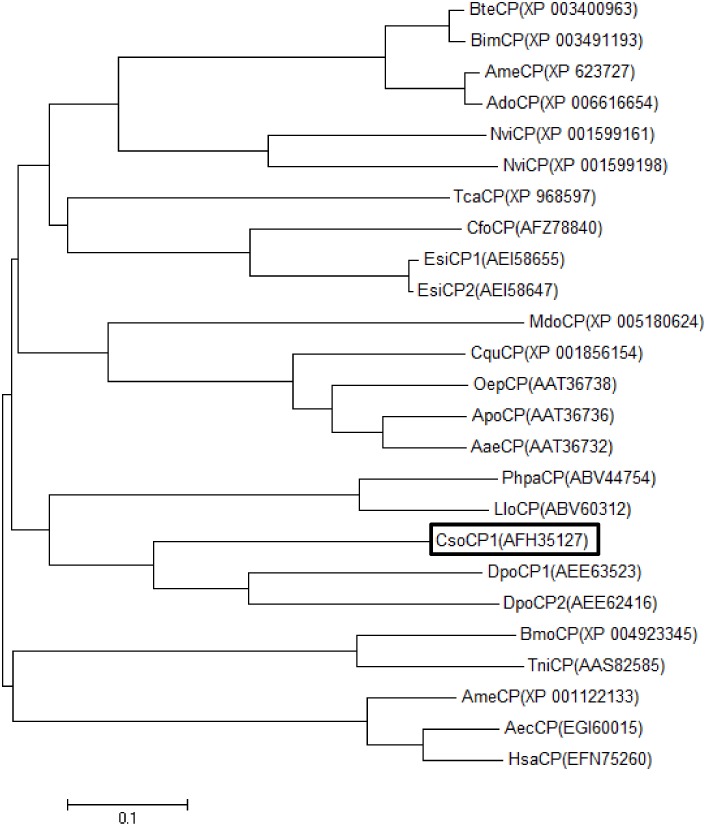
Phylogenetic analysis of carboxypeptidases from *C*. *sordidus* and other insect species (accession numbers are given). Phylogenetic analysis was conducted in MEGA6.0 using the Neighbor-joining method. Positions containing alignment gaps and missing data were eliminated only with pairwise deletion. Amino acid sequences of carboxypeptidases used for this analysis were *C*. *sordidus* (CsoCP), *D*. *ponderosae* (DpoCP), *Eupolyphaga sinensis* (EsiCP), *Nasonia vitripennis* (NviCP), *Coptotermes formosanus* (CfoCP), *Bombus terrestris* (BteCP), *Bombus impatiens* (BimCP), *T*.*castaneum* (TcaCP), *Apis mellifera* (AmeCP), *A*. *dorsata* (AdoCP), *Phlebotomus papatasi* (PhpaCP), *Aedes polynesiensis* (ApoCP), *Aedes aegypti* (AaeCP), *Lutzomyia longipalpis* (LloCP), *Culex quinquefasciatus* (CquCP), *N*. *vitripennis* (NviCP), *Bombyx mori* (BmoCP), *Acromyrmex echinatior* (AecCP), *Ochlerotatus epactius* (OepCP), *Musca domestica* (MdoCP), *Harpegnathos saltator* (HsaCP), *Trichoplusia ni* (TniCP).

A carboxypeptidase predicted protein (AFH35127.1), which was recently submitted to the GenBank from our group, shows 45–58% amino acid identity to other coleopteran carboxypeptidase-like proteins. Amino acid alignment of the predicted carboxypeptidase CsoCP1 with insect protease-like proteins is shown in the supplementary materials (S1 Fig). To determine the relatedness of the predicted proteinase-like proteins from the *C*. *sordidus* midgut transcriptome with other insect digestive enzymes, phylogenetic trees were constructed based on protein sequence. It was found that the carboxypeptidase predicted protein from *C*. *sordidus* (AFH35127.1) clustered together with two carboxypeptidase-like proteins from *D*. *ponderosae* (Scolytidae) (AEE63523 and AEE62416) (Fig 7).

### Semi-quantitative RT-PCR

**Fig 8:**
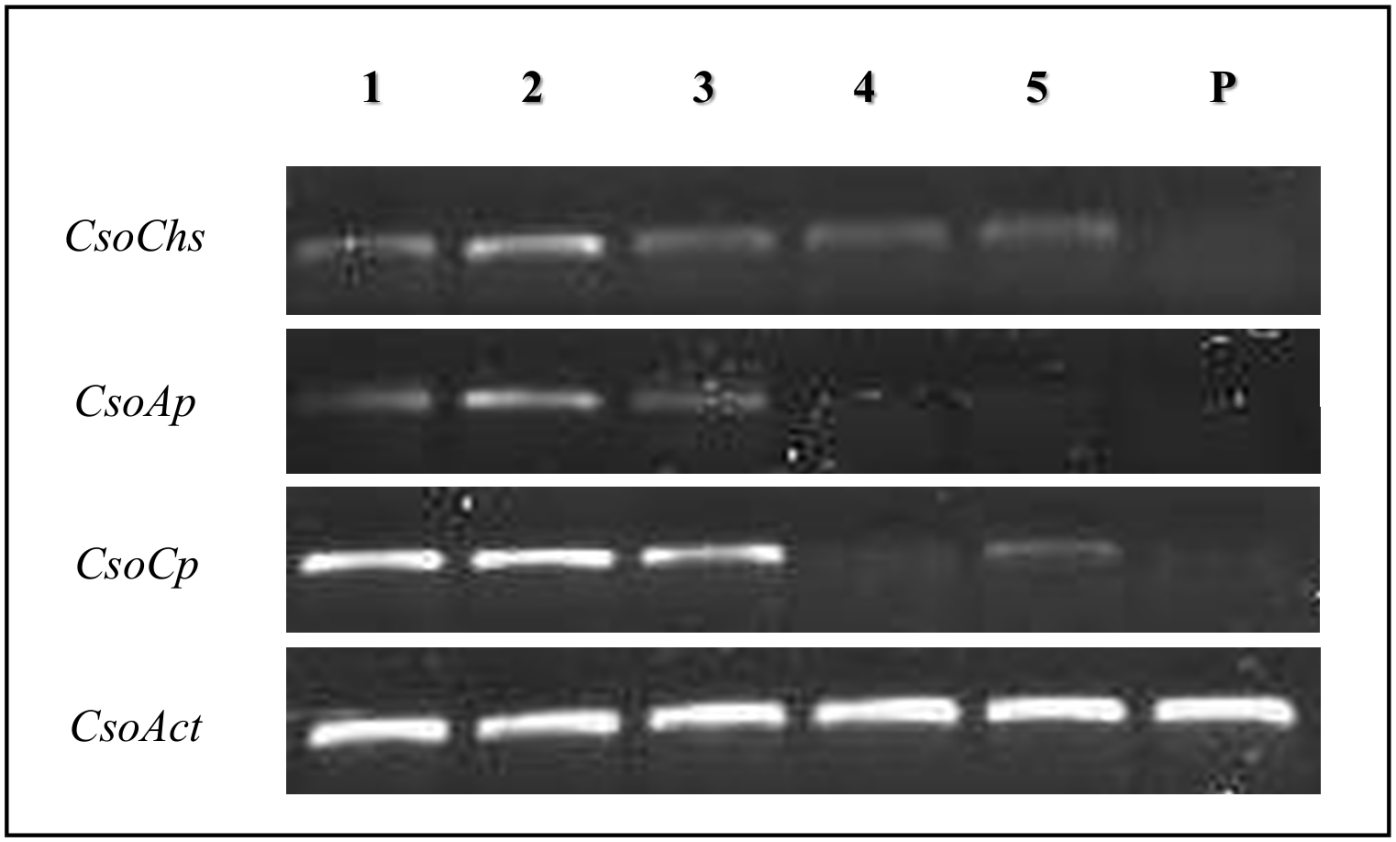
Semi-quantitative RT-PCR expression analysis of Carboxypeptidase (*CsoCp*), Chitin Synthase (*CsoChs*), Aminopeptidase (*CsoAp*) transcripts in five larval development and pupae stages of *C*. *sordidus*. Expression of the Actin Housekeeping gene (*CsoAct*) is shown as control. 1–5 represent first, second, third, fourth and fifth instar larvae while P is for pupae stage.

RT-PCR expression analysis was carried out to determine the expression profiles of specific enzyme-like transcripts in larval and pupae stages of *C*. *sordidus* (Fig 8). Results shows that expression of all protease-like transcripts were clearly visible through the first three larval stages. However, it was found that Chitin Synthase (*CsoChs*) transcript is expressing in all five larval development stages but not in pupae stage. In addition, none of evaluated transcripts was expressed in pupae stage. Just the control actin housekeeping gene (*CsoAct*) was clearly visible at all insect developmental stages.

## Discussion

Despite the enormous economic impact of the banana weevil *C*. *sordidus* on plantain crops worldwide [1], there is a general lack of transcriptome sequence data for this insect pest that could be used to examine traits of biological relevance that might be exploited for developing novel control methods. By using 454-based pyrosequencing, we obtained extensive sequence data providing an unprecedented opportunity for genomic research in an insect pest for which little genomic information is currently available. For example, transcriptome analysis in insects using 454-based pyrosequencing technologies have contributed significantly to the discovery of insect molecular markers (SNPs)[28], Bt receptors [20], immune responses [29, 30], insecticide targets and detoxifying enzymes [19, 31].

The transcriptomic data of the banana weevil *C*. *sordidus* that is presented here dramatically increases the number of *C*. *sordidus* midgut ESTs. For instance, the number of reported nucleotide sequences related to *C*. *sordidus* that were previously available represents only six genes (GenBank, February 15, 2016). This insect EST midgut collection provided by this study will allow the characterization of different genes not only for those closely related to other insect pests, but for many other coleopterans. A good example of this statement is found in the study of the molecular evolution of glycoside hydrolase (GH) genes in the western corn rootworm *Diabrotica virgifera virgifera* [32]. Results from this study have revealed the presence of three GH family genes (GH45, GH48, and GH28), which are found almost exclusively in Chrysomeloidea and Curculionoidea superfamilies, indicating the possibility of their acquisitions by horizontal gene transfer rather than simple vertical transmission. The transcriptomic analysis of the *C*. *sordidus* midgut provides an opportunity to identify genes unique to the *C*. *sordidus* midgut, thus providing an unprecedented opportunity for future insect specific management approaches. The 454-based deep pyrosequencing of the *C*. *sordidus* midgut transcriptome allowed the identification of contigs encoding proteins with functions strongly related not only to peritrophic membrane biosynthesis, membrane degradation and remodeling, detoxification, and immunity-related genes as well as defense against pathogens, but also to key digestive proteases involved with midgut physiology among many others (Table 2). Importantly, such genes could be targeted by using RNA interference (RNAi) which has been proposed as a novel control technology for other coleopterans. The banana weevil *C*. *sordidus*, like many other most insect species, can metabolize not only secondary plant chemicals but also insecticide-like chemicals, a metabolic process that includes a pool of detoxification enzymes such as cytochrome P450s, glutathione-S-transferase (EC 2.5.1.18), carboxylesterase (EC 3.1.1.1) and superoxide dismutase (EC 1.15.1.1) [19]. Transcripts encoding proteins linked to these detoxification enzyme families were found in the *C*. *sordidus* normalized midgut transcriptome. In total, 51 contigs were associated with xenobiotic metabolism. It has been reported that P450s represent a large superfamily of heme-containing monooxygenases that catalyze the metabolisms of exogenous and endogenous compounds [33]. A454-based transcriptomic analysis of greenhouse whitefly *Trialeurodes vaporariorum* identified 57 putative P450s [19]. However, it is possible that the number of these detoxification-related transcripts in the *C*. *sordidus* midgut transcriptome could be greater, especially the great variation of total number of P450 genes identified in different insect species [34] and availability of full length of some genes related to detoxification in this database, which could be a valuable prospect to be explored in future, this in turn will facilitate a better understanding of the role of these genes in xenobiotic metabolism and to evaluate the possibility of targeting some of them by using RNAi silencing technology.

As presented in Table 2, the most abundant uncovered protease-like transcripts in the *C*. *sordidus* midgut transcriptome are cysteine proteinases, serine proteinases, aminopeptidases and carboxypeptidases, indicating the widespread distribution of these protease-like genes in the *C*. *sordidus* midgut. It is well known that proteases are hydrolytic enzymes that are involved in many important roles in insect physiology from protein digestion to polyphenol oxidase activation [35]. The abundance of protease-like transcripts in the *C*. *sordidus* midgut transcriptome, as well as the expression of some of these specific transcripts as presented in results session of this manuscript, shows that the development of the banana weevil is extremely dependent on proteolytic enzymes indicating that those genes could represent a good target for RNAi-based technologies. In addition, the finding of the expression of the specific Chitin Synthase (*CsoChs*) transcript through all five larval development stages represent a strong evidence of the importance of these remodeling-like genes in insect metabolism. It is well known that chitin is not only the principal component of the arthropod cuticle, but also an integral part of peritrophic matrices [36], thus chitin synthesis is essential for insect development and survival and a potential target for RNA-based silencing technology (RNAi). In this context, previous research works have showed that RNAi-mediated down-regulation of *T*. *castaneum* CHS genes results in the reduction of chitin content [37].

Cysteine proteinases (EC 3.4.22) are digestive enzymes that have been isolated and partially characterized and which are widely distributed among many coleopteran species [38, 39]. Despite their importance in insect digestion, many of these protease-like enzymes remain poorly understood for their molecular functions. It is well known that the study of insect digestive enzymes has often focused on aminopeptidase-like enzymes due to the fact that this group of digestive proteases may act as natural receptors for Bt endotoxins [40, 41]. In fact, the insect midgut has become the primary target for both Bt-derived insecticides and a Bt alternative for pest control of *Chrysomela tremulae* [20]. The physiological role of these digestive-like enzymes in herbivorous insects like *C*. *sordidus* is to participate actively in the digestion of proteins. These enzymes cleave single amino acid residue from the N-terminus of proteins, which represents one of the most abundant compounds that are currently found in plant tissues. It is known that the expression level of proteases in insect guts depends on the protein content of the plant tissue that the insect uses as the main food source [35]. It is also important to point out that carboxypeptidases, with 22 contigs found in the *C*. *sordidus* transcriptome, represents an important group of peptidases in the banana weevil midgut. The lack of nucleotide sequences in the GenBank for this specific group of insect digestive enzymes (1 from our transcriptome data (AFH35127.1), as well as for many other gene sequences, will facilitate future research approaches that focus on *C*. *sordidus* peptidases and proteases. In addition, genes encoding proteinase inhibitors (PI) can represent a valuable alternative for control of insect pests when considering their inclusion into plant genomes using transgenic approaches [42]. Serine proteases are a group of digestive enzymes that are widely distributed in animals and microorganisms [43], playing key roles in many biological processes. As in *C*. *sordidus*, it has been also reported that many other insect species contains serine-type proteinases in their intestinal tract, allowing the insect to digest proteins that are naturally found in their food [44]. It has been observed that insects with alkaline midgut pH usually show higher serine proteinase activity [45], which are more active at neutral to alkaline pH, the condition of many lepidopteran insects. However, coleopteran insects that have a more acid pH in the digestive tract rely on cysteine or aspartic proteinases, which have better enzymatic activity at acidic pH [45].

Transcriptomic analysis of the *C*. *sordidus* genes involved in the insect immune and defense response led to the identification of C-type lectins, a protein family that has diverse functions, such as pathogen recognition and neutralization [46]. In the *C*. *sordidus* larval midgut EST database, the most abundant contig associated with immune response are C-type lectins followed by putative serine proteinase inhibitors or serpins. Similar results were found in the *Plutella xylostella* larval midgut transcriptome [47]. Results presented in this report represent the first transcriptomic analysis of the banana weevil *C*. *sordidus*, the most invasive and destructive pest of banana and plantain worldwide. This analysis has not only dramatically increased the number of known genes for this insect pest but it has also allowed the identification of novel gene sequences that are expressed in the intestinal tract providing a valuable source of information for understanding larval physiology and for identifying potential targets and management approaches for this insect pest or even as an important source of cDNAs in genome annotation. In addition, this transcriptome data adds to other research work focused on insect genome sequencing projects [48–50].

## Sequence Submission

The raw data obtained by 454-based pyrosequencing was submitted to the Short Read Archive database at NCBI (http://www.ncbi.nlm.nih.gov/guide/howto/submit-data/) (Accession SRP#: SRP061782).

## Supporting Information

S1 Fig Amino acid alignment of a predicted *C*. *sordidus* carboxypeptidase with known insect carboxypeptidase-like genes. An asterisk (*) indicates identical residues, semicolon (:) indicates highly conserved substitutions and a period (.) indicates semi-conserved substitutions. Dashes represent gaps introduced to preserve alignment. Species and accession numbers included in the alignment were *C*. *sordidus* (AFH35127), *D*. *ponderosae* (AEE63523), *P*. *papatasi* (ABV44754), and *L*. *longipalpis* (ABV60312).
(TIF)
