# Supplementary figures and images for: Pyrosequencing the Midgut Transcriptome of the Banana Weevil Cosmopolites sordidus (Germar) (Coleoptera: Curculionidae) Reveals Multiple Protease-Like Transcripts
Source: PLoS One. 2016 Mar 7;11(3):e0151001. doi: 10.1371/journal.pone.0151001 (PMC4780788; doi:10.1371/journal.pone.0151001)

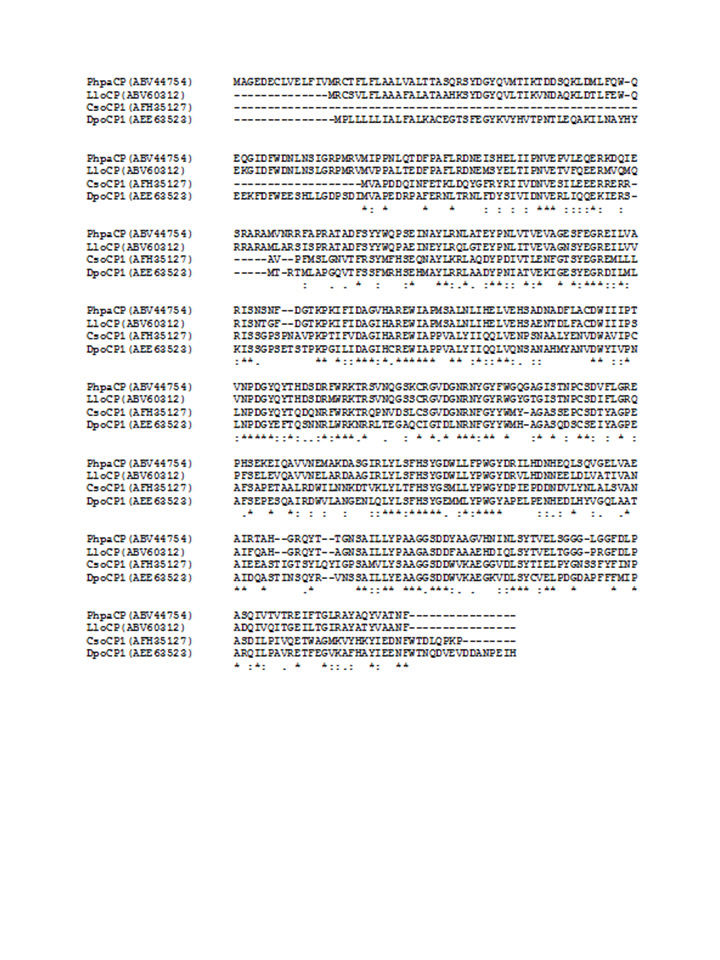

Supplement: S1 Fig — An asterisk (*) indicates identical residues, semicolon (:) indicates highly conserved substitutions and a period (.) indicates semi-conserved substitutions. Dashes represent gaps introduced to preserve alignment. Species and accession numbers included in the alignment were C. sordidus (AFH35127), D. ponderosae (AEE63523), P. papatasi (ABV44754), and L. longipalpis (ABV60312). (TIF) [file pone.0151001.s001.TIF]
